# Spatial Dimensions of Dengue Virus Transmission across Interepidemic and Epidemic Periods in Iquitos, Peru (1999–2003)

**DOI:** 10.1371/journal.pntd.0001472

**Published:** 2012-02-21

**Authors:** Kelly A. Liebman, Steven T. Stoddard, Amy C. Morrison, Claudio Rocha, Sharon Minnick, Moises Sihuincha, Kevin L. Russell, James G. Olson, Patrick J. Blair, Douglas M. Watts, Tadeusz Kochel, Thomas W. Scott

**Affiliations:** 1 Department of Entomology, University of California Davis, Davis, California, United States of America; 2 Fogarty International Center, National Institutes of Health, Bethesda, Maryland, United States of America; 3 Naval Medical Research Center Detachment, Washington D.C., United States of America; 4 Hospital de Apoyo de Iquitos, Región Loreto, Perú; 5 University of Texas at El Paso, El Paso, Texas, United States of America; Centers for Disease Control and Prevention, United States of America

## Abstract

**Background:**

Knowledge of spatial patterns of dengue virus (DENV) infection is important for understanding transmission dynamics and guiding effective disease prevention strategies. Because movement of infected humans and mosquito vectors plays a role in the spread and persistence of virus, spatial dimensions of transmission can range from small household foci to large community clusters. Current understanding is limited because past analyses emphasized clinically apparent illness and did not account for the potentially large proportion of inapparent infections. In this study we analyzed both clinically apparent and overall infections to determine the extent of clustering among human DENV infections.

**Methodology/Principal Findings:**

We conducted spatial analyses at global and local scales, using acute case and seroconversion data from a prospective longitudinal cohort in Iquitos, Peru, from 1999–2003. Our study began during a period of interepidemic DENV-1 and DENV-2 transmission and transitioned to epidemic DENV-3 transmission. Infection status was determined by seroconversion based on plaque neutralization testing of sequential blood samples taken at approximately six-month intervals, with date of infection assigned as the middate between paired samples. Each year was divided into three distinct seasonal periods of DENV transmission. Spatial heterogeneity was detected in baseline seroprevalence for DENV-1 and DENV-2. Cumulative DENV-3 seroprevalence calculated by trimester from 2001–2003 was spatially similar to preexisting DENV-1 and DENV-2 seroprevalence. Global clustering (case-control Ripley's K statistic) appeared at radii of ∼200–800 m. Local analyses (Kuldorf spatial scan statistic) identified eight DENV-1 and 15 DENV-3 clusters from 1999–2003. The number of seroconversions per cluster ranged from 3–34 with radii from zero (a single household) to 750 m; 65% of clusters had radii >100 m. No clustering was detected among clinically apparent infections.

**Conclusions/Significance:**

Seroprevalence of previously circulating DENV serotypes can be a predictor of transmission risk for a different invading serotype and, thus, identify targets for strategically placed surveillance and intervention. Seroprevalence of a specific serotype is also important, but does not preclude other contributing factors, such as mosquito density, in determining where transmission of that virus will occur. Regardless of the epidemiological context or virus serotype, human movement appears to be an important factor in defining the spatial dimensions of DENV transmission and, thus, should be considered in the design and evaluation of surveillance and intervention strategies.

## Author Summary

To target prevention and control strategies for dengue fever, it is essential to understand how the virus travels through the city. We report spatial analyses of dengue infections from a study monitoring school children and adult family members for dengue infection at six-month intervals from 1999–2003, in the Amazonian city of Iquitos, Peru. At the beginning of the study, only DENV serotypes 1 and 2 were circulating. Clusters of infections of these two viruses were concentrated in the northern region of the city, where mosquito indices and previous DENV infection were both high. In 2002, DENV-3 invaded the city, replacing DENV-1 and -2 as the dominant strain. During the invasion process, the virus spread rapidly across the city, at low levels. After this initial phase, clusters of infection appeared first in the northern region of the city, where clusters of DENV-1 and DENV-2 had occurred in prior years. Most of the clusters we identified had radii >100 meters, indicating that targeted or reactive treatment of these high-risk areas might be an effective proactive intervention strategy. Our results also help explain why vector control within 100 m of a dengue case is often not successful for large-scale disease prevention.

## Introduction

Dengue viruses (DENVs) cause more human morbidity and mortality worldwide than any other arthropod-borne virus [1], [2]. The principal vector is *Aedes aegypti*, a highly anthropophilic mosquito with relatively short dispersal tendencies that is known to bite people primarily during daylight hours as they engage in their daily activity patterns [3], [4], [5]. Interactions between relatively mobile humans and relatively sedentary mosquitoes are processes that underlie the dynamics of DENV transmission through space and time. Human movement can transport virus across small (households and neighborhoods) and large scales (village, city, country, and international) [6], [7], [8]. The contribution of infected female mosquitoes is restricted to short-range flight dispersal (household, neighborhood) [3], [9], [10], [11], [12], [13]. At small scales, several investigators have described dengue cases clustered within a household or neighboring houses [14], [15], [16], [17], [18], [19], [20]. The spatial dimension of DENV transmission beyond this very local scale of a household and its neighbors has been difficult to measure and thus a challenge to define [6], [21], [22]. One limitation is that previous investigations tended to focus on individuals with detectable dengue disease (fever and more severe illness), which represents only a fraction of all infections because, typically, a significant portion of human dengue infections are inapparent [23], [24], [25].

Transmission patterns of DENV in a geographic area are influenced by complex immunological interactions among the four closely related, antigenically distinct dengue viruses (DENV-1, DENV-2, DENV-3 and DENV-4) that make up the DENV complex [26]. Infection with one or more serotype(s) can result in a range of clinical outcomes, from asymptomatic infection to classic dengue fever, to more serious dengue hemorrhagic fever (DHF) and dengue shock syndrome (DSS). Because serotypes are antigenically distinct, primary infection confers lifelong homologous immunity, whereas heterologous cross-protection is short-lived [1], [27], [28], [29]. In many parts of Southeast Asia, especially in large urban centers, endemic transmission of all four serotypes occurs and broad scale, super-annual oscillations in dengue incidence have been reported [30]. In contrast, in many parts of Central and South America, epidemic transmission over the past three decades has occurred in distinct waves that are associated with invasion and amplification of single DENV serotypes or genotypes [31]. In those settings, endemic transmission patterns can be influenced by ambient levels of serotype-specific herd immunity and epidemic transmission can occur when a novel virus serotype enters a region where the majority of the population is immunologically naïve [17].

Since the re-introduction of DENV-1 into Iquitos during 1990, the city has experienced continuous DENV transmission with major epidemics occurring in association with novel serotype introductions. DENV-1 was introduced in 1991 and DENV-2 in 1995 [32], [33], [34]. By 2000, DENV-1 and DENV-2 were being transmitted at low, consistent levels from year to year. DENV-3 was then introduced in 2001 [35] and by late 2002 had replaced DENV-1 and DENV-2 as the dominant circulating serotype, causing a major outbreak of febrile illness. The local Peruvian Ministry of Health (PMoH) reacted by implementing household-level mosquito interventions (i.e., spraying insecticide inside houses), which appears to have truncated the epidemic [25].

To more carefully delineate patterns of transmission in Iquitos, we examined serological data from a city-wide, long-term, prospective, longitudinal study to describe and compare the spatial dimensions of DENV transmission across periods that transitioned from relatively low to high force of infection; i.e., from interepidemic to epidemic transmission. We compared clustering of infections (global and local) to test the hypothesis that the invasion of a new virus serotype or genotype follows a specific pattern: rapid, broad scale geographic spread at low levels [25], followed by large clusters of increased force of infection in predictable geographic regions. We also compared clustering of acute cases, as defined by Morrison *et al* 2001 [25]. At distances less than 100 m, we suspect that both mosquitoes and humans participate in virus transmission [3], , but beyond 100 m, it is likely primarily humans that define the spatial dimensions of the clusters. Previous research indicates that human movement often occurs well beyond a 100 m radius from their home (Vazquez Prokopec, Paz Soldan, Elder, Stoddard unpublished). Our results provide details on the dynamics of DENV invasion and establishment, have implications for evaluating intervention strategies, can be used to enhance dengue surveillance and prevention programs, and may be applicable to an improved understanding of transmission dynamics of other mosquito-borne pathogens.

## Methods

### Human Use Statement

The study protocol was approved by the University of California, Davis (Protocol 2220210788-4(994054), Instituto Nacional de Salud, and Naval Medical Research Center (Protocol #NMRCD.2001.0008 (DoD 31574) Institutional Review Boards in compliance with all Federal regulations governing the protection of human subjects. STROBE checklist included in supporting information (Checklist S1).

### Study area and population

**Figure 1:**
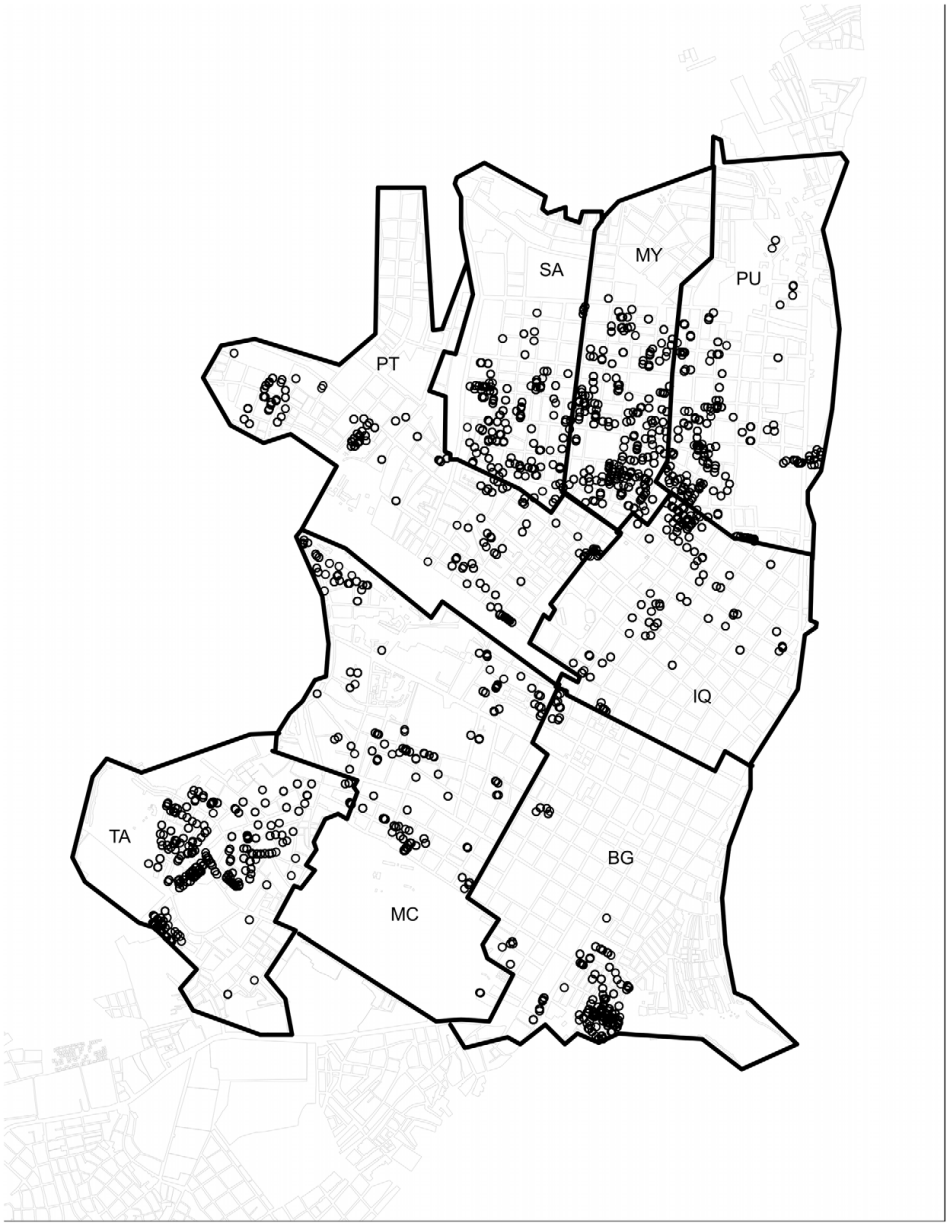
Distribution of cohort participant houses throughout the 8 zones of Iquitos. Each circle marks the location of a participant home.

Our cohort study was conducted in Iquitos, an isolated city of ∼400,000 people in the northeastern Amazon Basin portion of Peru. We divided the city into eight distinct geographical zones for our study (described in detail in Morrison *et al* 2004 [36]). The population consists of an approximate 1∶1 sex ratio, and about 36% of the population < = 17 years of age (http://desa.inei.gob.pe/).Written informed consent was obtained from participants older than 17 years and from parents of participants younger than 18. In addition, assent was obtained from participants 8–17 years of age. If participants were unable to read and sign the consent form, oral consent was obtained and documented. Blood samples were obtained from participants between January 1999 and August 2003, as described in Morrison *et al* 2010 [25] (Figure 1). Serum samples were tested for antibody to DENV serotypes based on plaque reduction neutralization test (PRNT) as described previously [25]. Briefly, heat-inactivated sera were incubated with DENV (DENV-1:16007; DENV-2: 16681; DENV-3: IQT1728) prior to inoculation onto BHK-21 cells. The level of neutralization used for the cutoff was PRNT70, with cutoff dilutions of 1∶60, 1∶80 and 1∶60 for DENV-1, DENV-2 and DENV-3, respectively. For more details, see Morrison *et al* 2010 [25].

### Extension of Morrison *et al* 2010

Morrison *et al* 2001 [25] provide a detailed description of overall patterns of DENV transmission in Iquitos from 1999–2005. In that study, the authors described data collected through active school-based surveillance and a longitudinal cohort, showing increasing seroprevalence of DENV-3 throughout the study. Here we extend these observational results with a more detailed analysis of spatial patterns in human infection.

### Cases and controls

Infections (i.e., cases) for our analyses below were defined as participants who showed evidence of serotype-specific seroconversion between two paired blood samples, based on PRNT. Controls were defined as susceptible, seronegative participants who had no serological evidence of serotype-specific infection between paired blood samples.

Both cases and controls were limited to participants whose paired blood samples were less than 242 days apart, corresponding to the 75% quartile of the distribution of intervals. Analyses of the dataset were run independently for each distinct serotype. A trimester-specific dataset was constructed containing all cases and controls with middates falling within that period. Yearly trimester intervals (Jan–Apr: end of the high transmission season, May–Aug: low transmission season, and Sept–Dec: beginning of the high transmission season) were chosen for analyses because they capture intra-annual variation in the Iquitos DENV transmission season. Although there is variation from year to year, the high DENV transmission season in Iquitos generally occurs from Sept–Apr, unless emergency vector control interventions decrease transmission prior to April. In general, consistently lower rates of transmission were observed between May and July [25], [31].

### Seroprevalence

At the initiation of the cohort (Jan 1999), DENV-1 and DENV-2 were circulating at low levels throughout Iquitos. The baseline serostatus of participants was determined over the eight geographic zones using samples collected between January and October of 1999 [25].

To measure the prevalence of antibodies for DENV-3 throughout the course of its invasion into Iquitos, overall participant serostatus was calculated using the most recent sample taken from each participant, beginning in Aug 2002. As with baseline seroprevalence rates, cumulative seroprevalence of DENV-3 was estimated for the eight Iquitos geographical regions from Jan 2002–May 2003.

To determine if the invasion of DENV-3 was positively correlated with the baseline seroprevalence of DENV-1/DENV-2 across the city prior to its introduction, we conducted a Spearman Rank correlation test, comparing the DENV-3 seroprevalence by zone in each trimester to the baseline DENV-1/DENV-2 seroprevalence. For this analysis, as the ρ coefficient approaches −1 or 1 the two patterns are more negatively or positively correlated, respectively.

**Figure 2:**
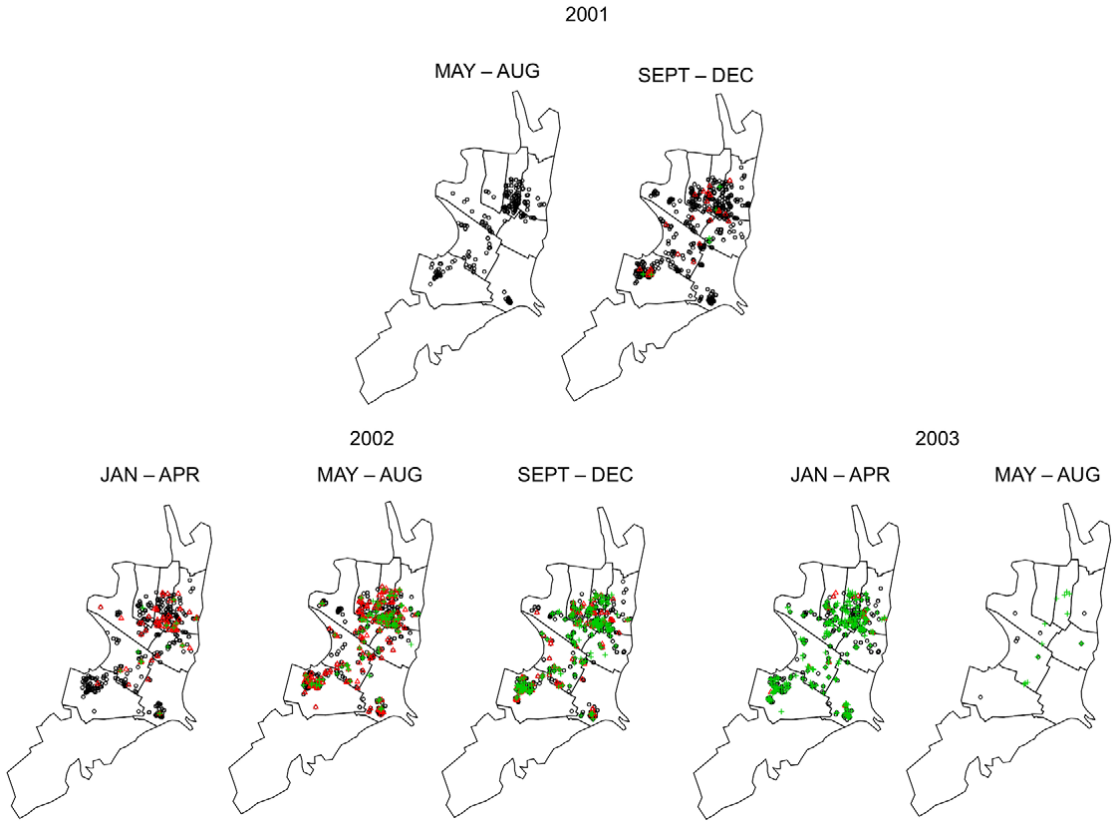
Distribution of all participants sampled during 4-month interval. Black = DENV-3 susceptible, red = DENV-3 infected, green = DENV-3 immune.

Sampling effort across the eight geographical zones varied, due primarily to the location of commercial versus residential areas of the city (Figure 2). To visualize the spatial pattern of accumulating seroprevalence throughout the study period, we used spatial kernels based on the intensity of points in the study area for each trimester. Points were the coordinates of participants' homes. We first generated a kernel for the pattern of all participants with a middate in a given trimester. Similarly, we then generated a kernel of individuals immune to DENV-3 (presenting with antibody in the first blood sample). Because of the nature of kernel estimation, estimates where there are no points nearby tend to be small. Calculating the ratio of immune to all participants to calculate seroprevalence would, therefore, result in artificially high estimates when both of these values were small. For the kernel of immune individuals, we thus set all values falling into the first quartile to zero in order to avoid gross overestimates of seroprevalence. All kernels were estimated assuming a standard, isotropic Gaussian kernel with fixed bandwidth (σ = 175), which was determined by visual inspection of the results. All analyses were conducted using the SpatStat package of the R Statistical Computing Environment [37].

### Spatial analyses

To determine the spatial dynamics of DENV transmission, we examined patterns of both global and local clustering. The global analysis was used to examine the spatial pattern of seroconversions within a defined reference window (the eight zones) and, by comparison to the pattern of controls, to test the hypothesis that seroconversions clustered in space. On the other hand, local clustering uses a specific spatial window to look for the over-dispersion of cases in comparison to controls within that window. This window of delimited radius X moves across the geocoded data for each time interval and indicates when cases appear in greater numbers than expected.

### Global pattern clustering

Global patterns of dengue seroconversions were analyzed within each trimester using a case-control Ripley’s K statistic [38]. When used within a Monte Carlo framework, this allows for inferences to be made about the structure of a point pattern. To test the hypothesis that dengue seroconversions clustered in space, we simulated an inhomogeneous K function (because of the heterogeneous sampling pattern) comparing random samples of control points to cases of dengue infection in each time interval using the participants’ home as their spatial reference. Simulations were conducted by resampling the control points with replacement 99 times to estimate the minimum and maximum K functions for susceptibles, generating a 99% probability envelope. The number of control points sampled was always equivalent to the number of cases. Within this framework, deviation of the K function for DENV seroconversions outside of the probability envelope at a specific distance was evidence of global clustering (if above the envelope) or repulsion (if below the envelope) and supports rejection of the null hypothesis that the patterns are equivalent at a probability level of 0.01 [37].

### Local pattern clustering

Local clustering of serotype-specific DENV seroconversions was detected using the Kuldorff spatial scan statistic [39], [40] with SaTScan software verson 8.1.1 (http://www.satscan.org). This method uses a retrospective spatial Bernoulli probability model to detect significant spatial clustering of cases. Briefly, using the Bernoulli probability distribution with Monte Carlo simulation repeated 999 times, the model tests the statistical likelihood of the distributions of cases in relation to controls for a moving circular spatial window, determining if a greater than expected number of cases occurred in an area. We assessed patterns across several different spatial radii (100, 300, 600 and 900 m) for each serotype-specific trimester dataset. Spatial scan analyses were only run for a specific serotype and trimester when more than two seroconversions were observed during that time period. In total, 92 analyses were conducted (32 for DENV-1, 32 for DENV-2, and 28 for DENV-3). Identical SaTScan analyses were run on 64 laboratory confirmed, acute, apparent DENV infections captured by active surveillance for febrile illness, as described previously (for details see Morrison *et al* [25] and Rocha *et al* [41]).

## Results

### Study population

A total of 3,110 participants met the inclusion criteria for our analyses. As described in Morrison *et al* 2010 [25], the majority of participants (n = 2,393, 76.93%) were under the age of 18. The number of males (n = 1,305, 41.96%) was less than that of females.

### Cases and Controls

Over the course of the study period, we detected a total 607 seroconversions to DENV (68 to DENV-1, 58 to DENV-2 and 481 to DENV-3; Table 1). The total number of controls for each serotype/trimester is presented in Table 1. The population included in the analyses varied by serotype for specific years and trimesters, due to changes in serotype-specific immunity over time.

**Table 1:** Total serotype-specific cases and controls by trimester.

DENV-1				
YEAR	TRIMESTER	CASES	CONTROLS	POPULATION
1999	MAY–AUG	3	285	288
2000	JAN–APR	10	599	609
2000	SEPT–DEC	6	352	358
2001	JAN–APR	9	296	305
2001	SEPT–DEC	8	289	297
2002	JAN–APR	16	271	287
2002	MAY–AUG	7	392	399
2002	SEPT–DEC	9	344	353

DENV-2				
YEAR	TRIMESTER	CASES	CONTROLS	POPULATION
1999	MAY–AUG	6	241	247
2000	JAN–APR	18	505	523
2000	MAY–AUG	4	294	298
2000	SEPT–DEC	9	280	289
2001	JAN–APR	3	237	240
2002	JAN–APR	3	236	239
2002	MAY–AUG	6	361	367
2002	SEPT–DEC	9	343	352

DENV-3				
YEAR	TRIMESTER	CASES	CONTROLS	POPULATION
2001	JAN–APR	4	747	751
2001	MAY–AUG	0	391	391
2001	SEPT–DEC	28	970	998
2002	JAN–APR	69	676	745
2002	MAY–AUG	233	977	1210
2002	SEPT–DEC	130	727	857
2003	JAN–APR	17	520	537

### Seroprevalence

**Figure 3:**
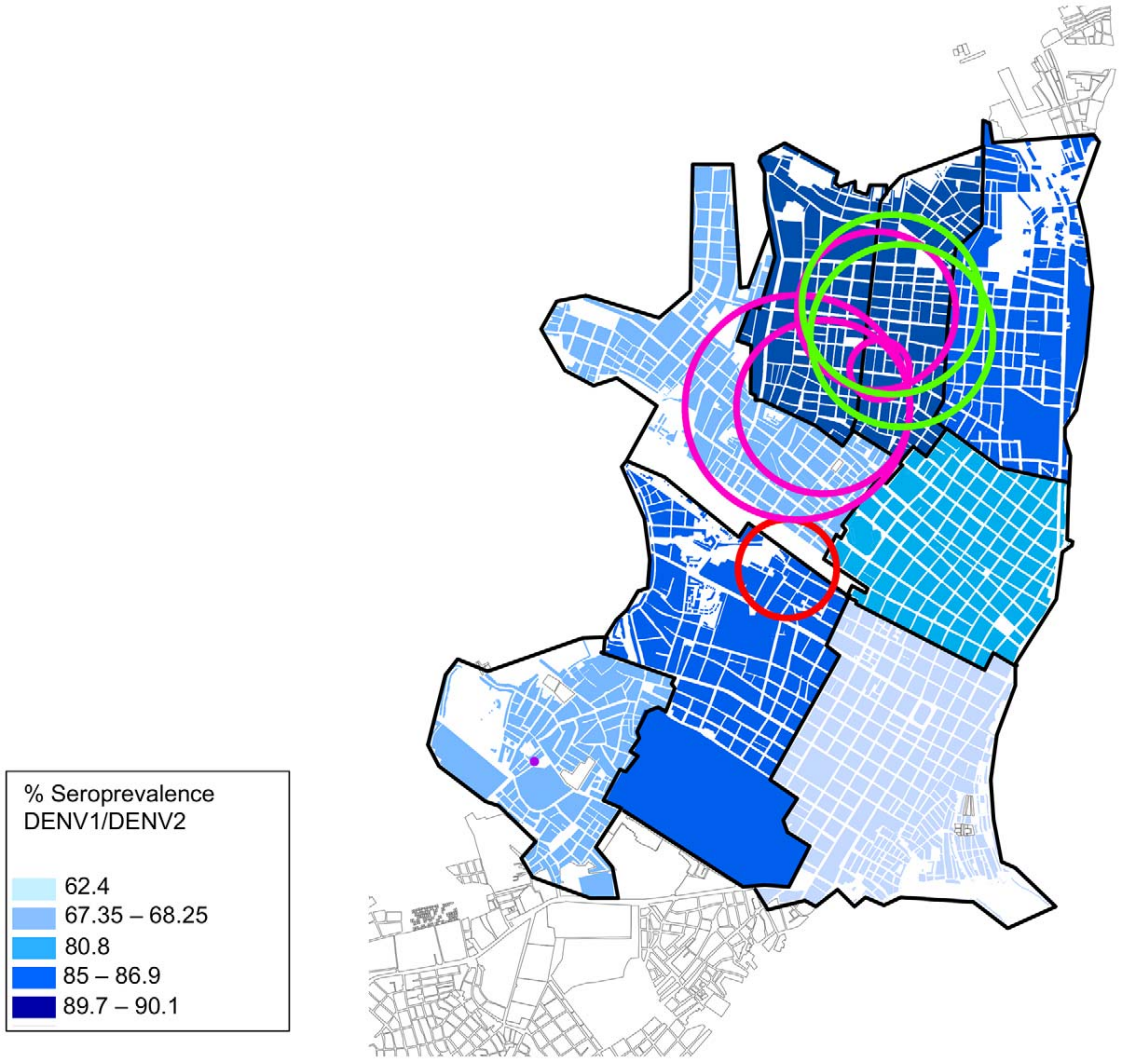
Seroprevalence of DEN-1 and DEN-2 in the 8 geographic zones of Iquitos in October 1999. Clusters of DENV-1 are indicated by colored circles (purple = May–Aug 1999; red = Jan–Apr 2000; pink = Sep–Dec 2001; green = Jan–Apr 2002). No significant clusters of DENV-2 were identified during the study period.

From samples taken between January and October 1999, a total of 1,999 participants met the inclusion criteria listed above. Of those, monotypic neutralizing antibodies (NtAbs) against DENV-1 and DENV-2 were detected in 12% (n = 240) and 14.7% (n = 294), respectively. A total of 1,054 participants (52.7%) had detectable NtAbs against both DENV-1 and DENV-2 at baseline, whereas 20.6% (n = 411) showed no evidence of prior dengue infection. These percentages are similar to those reported by Morrison *et al* 2010 [25], indicating that the population meeting the inclusion criteria was similar in serostatus to those of the overall cohort. When analyzed by zone, DENV-1 and DENV-2 seroprevalence show a distinct geographic structure (Figure 3), similar to that previously described by Morrison *et al* 2010 [25]. At the initiation of the study, the prevalence of DENV-3-specific antibodies was low (<5%), but rapidly increased city-wide over the course of the study with distinct differences between city zones.

**Figure 4:**
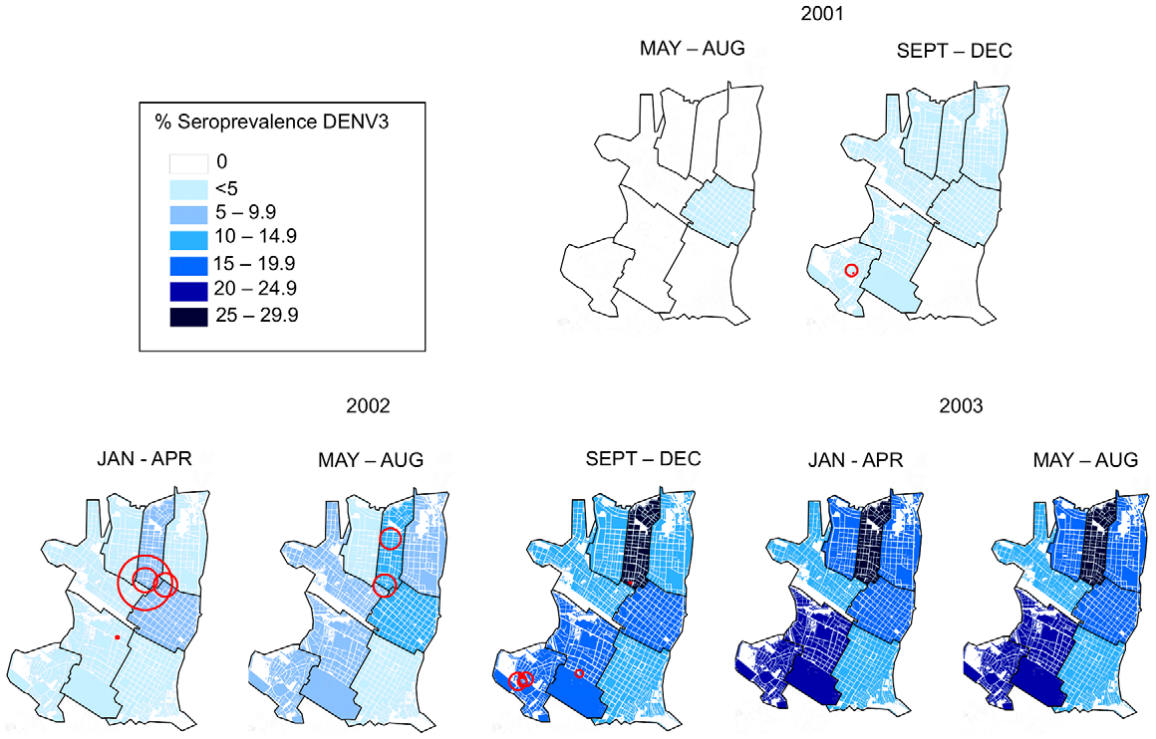
Seroprevalence of DEN-3 by trimester. Pink circles indicate significant clusters.

**Figure 5:**
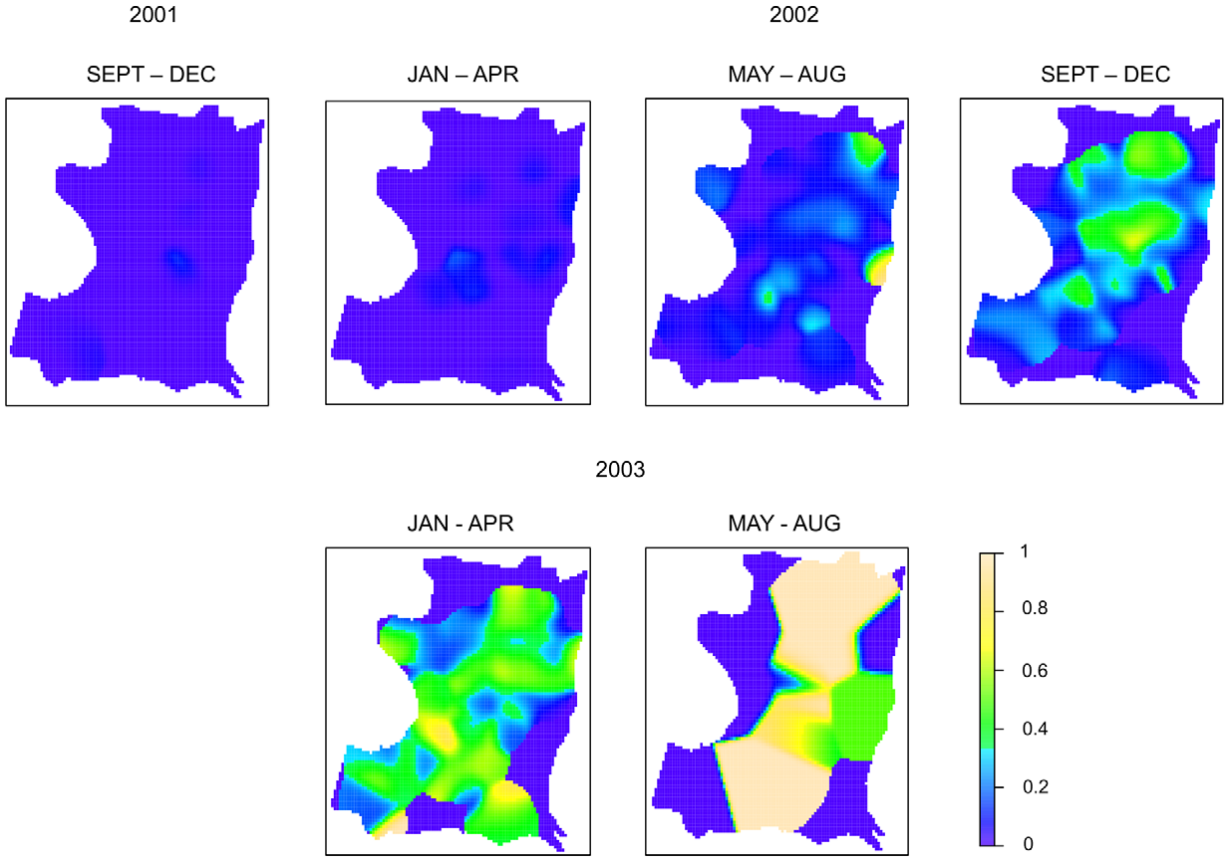
Seroprevalence kernel of DENV-3 by trimester.

Invasion of DENV-3 into Iquitos is clearly demonstrated by changes in temporal and geographic seroprevalence patterns (Figure 4, Figure 5, Table 2). Transmission was first detected in the city center (Iquitos [IQ]) during the May–Aug 2001 trimester (2001.2) and then was detected at rates of less than 5% in all but one of the eight geographic zones during the Sep–Dec 2001 trimester. Although seroprevalence rates remained low throughout the city during the first part of 2002, virus was concentrated in the northern zones of Maynas (MY) and IQ. As indicated by Morrison *et al* 2010 [25], other zones lagged behind MY and IQ, with most failing to reach the levels of seroconversion observed in MY, where the highest overall DENV transmission rates occurred.

**Table 2:** Seroprevalence rates DENV-3 by trimester in the 8 regional zones of Iquitos.

	2001		2002			2003	
ZONE	MAY–AUG	SEPT–DEC	JAN–APR	MAY–AUG	SEPT–DEC	JAN–APR	MAY–AUG
BG	0%	0%	2%	2%	12%	13%	13%
TA	0%	1%	3%	6%	18%	20%	20%
PT	0%	1%	3%	7%	13%	15%	15%
IQ	1%	2%	6%	11%	16%	16%	16%
PU	0%	1%	2%	7%	15%	19%	19%
MC	0%	1%	4%	7%	20%	22%	22%
MY	0%	1%	6%	12%	27%	28%	29%
SA	0%	1%	2%	3%	15%	16%	17%

The Spearman ρ correlation coefficient comparing the DENV-3 seroprevalence in the first trimester of 2002 with the baseline seroprevalence of DENV-1/DENV-2 is close to zero (0.24) and is not statistically significant (p = 0.58). This implies little to no correlation between the two patterns. The correlation between DENV-1/DENV-2 seroprevalence and DENV-3 seroprevalence increased in each subsequent trimester, with ρ = 0.48 (p = 0.24) and 0.62 (p = 0.12) in the second trimester and third trimesters of 2002, respectively. In the first trimester of 2003, ρ increased to 0.67 (p = 0.08). It is important to note that these data only follow the invasion of DENV-3 through its first two years in Iquitos. Due to a change in the sampling scheme after the 2002–2003 transmission season, we were unable to compare the seroprevalence data from the final cohort sample collected in 2005.

### Global patterns

**Figure 6:**
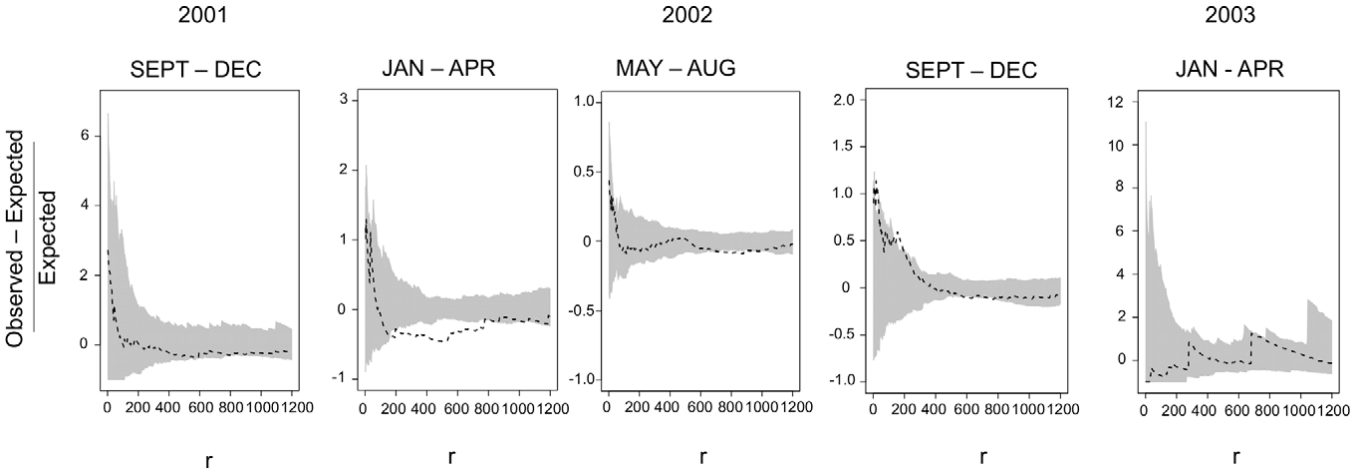
Monte Carlo K statistic. The difference between the observed and expected over the expected is plotted against distance, r. The grey envelope is the 99% probability envelope based on the pattern of controls and the black line is the K function ofor the cases (seroconversions). If the line strays above the envelope, this indicates clustering. If it strays below, this indicates repulsion or over dispersion.

To facilitate visual interpretation of the Monte Carlo K-function results, we plotted the ratio of the difference between the K functions for cases and controls over the K-function for controls. We failed to reject the null hypothesis that the pattern of cases was different from that of controls if the cases K function fell between the high and low control K functions (99% probability envelope). For DENV-1 and DENV-2, the number of seroconversions within any given trimester was small and always consistent with the pattern of controls. When considering all seroconversions within an epidemiological season, DENV-1 showed evidence of clustering at ∼200 m during the 2001–2002 season. For DENV-2 the pattern of seroconversions was more widely dispersed than expected during the 2000–2001 season at distances of ∼220 m–500 m. Overall, however, there was little evidence for spatial structuring of DENV-1 and DENV-2. In the case of DENV-3, we observed that during the first trimester when seroconversions occur (2001.3), the pattern of cases was no different from that of controls, providing evidence that this new serotype had rapidly spread within one trimester at low levels to all parts of the city (Figure 6). In the next trimester (2002.1), however, the pattern was highly over-dispersed relative to controls across a range of scales from 200 m to ∼700 m. Examination of the pattern of seroconversions was consistent with there being a focus in the northern region of the city in the MY zone with additional seroconversions radiating out from there. During the next trimester (2002.2) the pattern of cases again indicated overdispersion, but at broader scales ∼800 m. During the third trimester (2002.3), DENV-3 seroconversions appeared more clustered at small scales - ∼15 m and ∼180 m – possibly reflecting transmission among neighboring households and within neighborhoods. During the first trimester of 2003, after the PMoH intervention, the pattern of cases was no different than that of controls.

When analyzed by epidemiological season, DENV-3 seroconversions were highly clustered relative to expectation up to ∼100 m during the initial invasion phase during the 2002 season (Jul 1 2001–Jun 30 2002). For the 2003 season (Jul 1 2002–Jun 30 2003) cases were equivalent to controls. The fact that at a broader temporal scale (season) the pattern of seroconversions was clustered during the 2002 season, while the pattern by trimesters was over-dispersed, appears to reflect the existence of alternate, neighborhood-scale (∼200–800 m) foci occurring at different times during the invasion and establishment of the virus.

### Local patterns

From the 92 spatial scan analyses run, a total of 23 significant (p = <0.05) clusters were identified: eight for DENV-1 and 15 for DENV-3 (Table 3). Of these, 65% (n = 15) had radii greater than 100 m. No significant clusters were detected for DENV-2 during any trimester. Similarly, we detected no significant clusters of febrile cases captured by active surveillance.

**Table 3:** Significant clusters of DEN-1 and DEN-3 from 1999 through April of 2003.

CLUSTERS OF DENV-1						
Year	Trimester	Parameter (meters)	Cases	Controls	Radius (meters)	P-value
1999	MAY–AUG	100	3	0	8.7	< = .001
2000	JAN–APR	600	3	3	330	0.015
2001	SEPT–DEC	300	4	4	200	0.006
2001	SEPT–DEC	600	6	15	530	< = .001
2001	SEPT–DEC	600	5	17	580	0.015
2001	SEPT–DEC	900	6	23	750	0.008
2002	JAN–APR	600	8	23	600	0.032
2002	JAN–APR	900	10	35	610	0.01

CLUSTERS OF DENV-3						
Year	Trimester	Parameter (meters)	Cases	Controls	Radius (meters)	P-value
2001	SEPT–DEC	100	3	0	0	0.015
2001	SEPT–DEC	300	8	29	130	0.004
2002	JAN–APR	100	6	4	33	0.022
2002	JAN–APR	300	18	45	260	0.006
2002	JAN–APR	300	13	30	270	0.032
2002	JAN–APR	600	34	120	600	< = .001
2002	MAY–AUG	300	20	12	230	0.002
2002	MAY–AUG	300	31	43	250	0.011
2002	SEPT–DEC	100	21	14	98	< = .001
2002	SEPT–DEC	100	7	0	7.2	0.002
2002	SEPT–DEC	100	9	2	82	0.003
2002	SEPT–DEC	100	5	0	18	0.026
2002	SEPT–DEC	100	5	0	40	0.026
2002	SEPT–DEC	300	30	22	190	< = .001
2002	SEPT–DEC	300	33	48	150	< = .001

No significant clusters of DENV-2 were identified during the study period.

Clusters of seroconversions to DENV-1 were spread throughout the city, with different high-risk zones each trimester (Figure 3). No visual pattern appeared to link transmission clusters from one period to the next.

For epidemic DENV-3, however, a more apparent pattern emerged. As time passed, clusters concentrated in areas following the distinct pattern of seroprevalence detected for DENV-1 and DENV-2 during 1999 (Figure 3, Figure 4). Clusters began in the north of the city, where overall DENV seroprevalence rates were highest. For example, in the northern region of the city in the second trimester of 2002 statistically significant clusters are observed in MY, where seroprevalence rates increased from 12% in the second trimester of 2002 to 27% in the third. In contrast, in other zones with significant but overall lower DENV transmission, we were unable to identify statistically significant clustering.

## Discussion

Understanding the underlying spatial dimensions of DENV transmission in different epidemiological contexts will improve our understanding of virus transmission dynamics and aid in the design and deployment of preventative and emergency control measures. While we did not detect spatial clustering among people with clinically apparent DENV infections, all infections (i.e., apparent and inapparent) exhibited spatially distinct transmission patterns. Although details of DENV transmission differed between interepidemic and epidemic periods, we speculate that in Iquitos human movement is a unifying process in delimiting virus transmission across different epidemiologic contexts.

During the interepidemic period (1999–2001), DENV-1/DENV-2 incidence rates were highest in the southern regions of the city where overall seroprevalence was lowest [25]. Significant clustering of DENV-2 was not detected throughout this study, likely due to the fact that only 49 seroconversions were detected during the study period. However, we did detect significant clusters of DENV-1 seroconversions in northern regions of the city, where DENV seroprevalence was highest. Mosquito populations monitored throughout the study showed high adult *Ae. aegypti* indices in the northern zone of Maynas, where seroprevalence was also highest, indicating an elevated level of infection risk [25], [36].

Our results thus indicate that a positive relationship may exist between mosquito abundance and the risk of human DENV infection. At the levels we observed, herd immunity was insufficient to counteract risk from other factors, such as elevated mosquito exposure, which could serve as a catalyst for neighborhood-specific variation in DENV transmission. The relationship between entomological indices and DENV transmission has been difficult to define [21], [22]. Malariologists reported that increased infective mosquito bites per person per day [entomological inoculation rate (EIR)] correlates with higher malaria incidence in humans [42], [43], [44], [45]. A similar relationship has not, however, been established for DENV and *Ae. aegypti*, but is worth exploring in greater detail [46].

We did not identify spatial patterns among 64 people with overt illness, implying that defining spatial transmission patterns based solely on clinically apparent DENV infections can be misleading. Spatial patterns for seroconversions were more easily detected during the DENV-3 invasion than during the interepidemic period. After initially spreading rapidly and evenly at low rates throughout the city, distinct spatial patterns of DENV-3 transmission were detected. For example, MY, the first zone to experience clusters of DENV-3, also had the highest combined seroprevalence rates for DENV-1 and DENV-2 prior to the DENV-3 introduction. DENV-3 transmission started earlier and was higher overall in MY than in the rest of the city. This observation was consistent with there being underlying spatial heterogeneity of DENV transmission in Iquitos, perhaps associated with some aspect of the mosquito populations, that puts persons living in certain parts of the city at higher intrinsic risk of infection than people in other locations.

As DENV-3 invaded the city, its establishment closely followed the geographic patterns observed for seroprevalence of the DENV serotypes that preceded it. While the results of the correlation rank test were not statistically significant at a 0.05 level, the pattern of DENV-3 seroprevalence appeared to be converging on that of DENV-1/DENV-2. Had we maintained the same sampling scheme throughout the remainder of the DENV-3 invasion, we would anticipate the correlation of the patterns to become significant. Historic, geographically distinct seroprevalence patterns, therefore, may be useful for prioritizing dengue surveillance (i.e., infection in humans and/or mosquitoes) and prevention (i.e., mosquito control or vaccination). Preemptive intervention could be directed towards areas that are considered particularly susceptible to elevated risk of virus transmission and from which virus could spread to other locations. Focusing control on areas of high transmission may help diminish the impact of a novel virus invasion. The effect that targeting areas with high levels of transmission may have on transmission at broader geographic scales remains to be determined. Targeting areas that theoretically contribute most to transmission [47], [48] is attractive because it could be a more effective way to use limited resources for disease prevention than uniform application over large geographic areas [21], [22].

Transmission foci during epidemic and interepidemic periods had radii that exceeded 100 m in 65% of significant clusters, indicating that spatial dimensions for DENV transmission extended beyond the level of individual households and the flight range *Ae. aegypti* [3]. The relative role of humans versus mosquitoes in DENV movement within 100 m has not been resolved. At greater distances, human movement of virus appears to be an important factor in defining the spatial dimensions of DENV transmission regardless of the epidemiologic context; i.e., interepidemic and epidemic periods as well as during the invasion and establishment of a novel virus (Stoddard *et al* in prep).

Since the completion of our study, DENV-3 caused significant outbreaks of disease in 2004, 2006–2007, and again in Feb 2008 when the first isolates of DENV-4 in Iquitos were recovered from participants in a clinic-based fever study [31]. Following its introduction, DENV-4 became the main circulating virus throughout the city until late 2010 when an Asian-American strain of DENV-2 was introduced into Iquitos, causing a large city-wide outbreak [49], [50]. Analyses of the spatial dimensions of these two serotype introductions would be useful for determining whether the patterns remain consistent. Because subsequent epidemiological studies used distinct spatial designs and had distinct research objectives we are unable to include those data in this presentation, but we can confirm that MY was one of the first effected during both the DENV-4 and DENV-2 outbreaks (Scott and Morrison unpublished).

Our study had three notable limitations. First, since we only know the time interval during which infection occurred but not its exact date, we may not have identified all significant virus transmission clusters. DENV transmission and spread in a community can happen rapidly. Although drawing blood at six-month intervals is appropriate for the analysis of seroprevalence over time, a shorter temporal window between assessments of serostatus might improve resolution in spatial analyses of DENV invasion. This limitation might in part account for our inability to predict clusters in certain areas where seroprevalence rates quickly increased over time. This could occur because the period between two blood draws may span two trimesters. For instance if the first sample was taken November 30 2001 and the second April 28 2002, the middate would occur in the first trimester of 2002, whereas the actual infection might have occurred in the third trimester of 2001. Despite these limitations, our analyses still provide insights across all infections, including those that were inapparent. Second, because of our sampling scheme, we focused our spatial analyses of seroprevalence at the zone level. Dividing the cohort into smaller units (i.e., blocks) would not properly represent the population. Third, our school-based active surveillance sampling scheme may not have identified all acute DENV infections and thus contributed to the lack of detectable spatial patterns among clinically apparent infections. In subsequent cohort studies, we used a different sampling scheme in an effort to examine transmission patterns at smaller spatial scales.

Current World Health Organization (WHO) guidelines, which recommend vector control be administered to households within a 400 m radius of a dengue case, are consistent with the transmission clusters we detected [51]. Vector control at this scale in a city such as like Iquitos could, however, require that hundreds of houses be treated for each case. Consequently, in practice a radius of 100 m is often adopted. Even though small scale clustering is clearly important, our local and global analyses identified a significant proportion of spatial clusters that extended well beyond 100 m. Our results, therefore, provide an additional explanation for why vector control within 100 m of a dengue case has been less successful than desired for larger scale disease prevention [21], [22].

Results from our cluster analyses indicate that if areas of primary invasion and/or elevated amplification can be treated based on historical patterns of transmission (i.e., seroprevalence) either *a priori* or reactively, it may be feasible to block virus dispersal. The majority (93%) of clusters we detected had radii <400 m, indicating that neighborhoods where initial cases are identified could be intervention targets. A substantial challenge for reactive control will be the need to quickly apply the intervention at the correct locations during the early phases of invasion when most human infections are clinically inapparent and therefore difficult to detect in disease-based surveillance systems [24]. Alternatively, it may be more productive to focus on characterizing historical seroprevalence patterns as a means for prioritizing spatially targeted surveillance and preemptive intervention. The DENV-3 Iquitos invasion occurred more rapidly and at larger scales than we were able to quantify with our longitudinal cohort study. Future research could explore the preemptive implementation of surveillance and control in areas where the highest initial incidence was previously detected, and from where virus may disseminate to the other parts of the city.

## Supporting Information

Checklist S1 **STROBE checklist.**
(DOC)
